# Impact of Urate Level on Cardiovascular Risk in Allopurinol Treated Patients. A Nested Case-Control Study

**DOI:** 10.1371/journal.pone.0146172

**Published:** 2016-01-11

**Authors:** Kasper Søltoft Larsen, Anton Pottegård, Hanne M. Lindegaard, Jesper Hallas

**Affiliations:** 1 Clinical Pharmacology, Department of Public Health, University of Southern Denmark, JB Winsloewvej 19.2, DK-5000, Odense C, Denmark; 2 Department of Clinical Chemistry & Pharmacology, Odense University Hospital, JB Winsloewvej 19.2, DK-5000, Odense C, Denmark; 3 Department of Rheumatology, Odense University Hospital, Sdr. Boulevard 29, DK-5000, Odense C, Denmark; Kagoshima University Graduate School of Medical and Dental Sciences, JAPAN

## Abstract

**Background:**

Gout gives rise to increased risk of cardiovascular events. Gout attacks can be effectively prevented with urate lowering drugs, and allopurinol potentially reduces cardiovascular risk. What target level of urate is required to reduce cardiovascular risk is not known.

**Objectives:**

To investigate the effect of achieving target plasma urate with allopurinol on cardiovascular outcomes in a case-control study nested within long-term users of allopurinol.

**Methods:**

We identified long-term users of allopurinol in Funen County, Denmark. Among these, we identified all cases of cardiovascular events and sampled 4 controls to each case from the same population. The cases and controls were compared with respect to whether they reached a urate target below 0.36 mmol/l on allopurinol. The derived odds ratios were controlled for potential confounders available from data on prescriptions, laboratory values and in- and outpatient contacts.

**Results:**

No association between treatment-to-target urate level and cardiovascular events were found (adjusted odds ratio of 1.01, 95% confidence interval 0.79–1.28). No significant effect was seen in any subgroup defined by age, gender, renal function, allopurinol dose or the achieved urate level. Overall, the doses of allopurinol used in this study were low (mean ≈ 140 mg/day).

**Conclusion:**

We were unable to demonstrate a link between achieved urate level in patients treated with allopurinol and risk of cardiovascular events. Possible explanations include that allopurinol doses higher than those used in this study are required to achieve cardiovascular risk reduction or that the cardiovascular effect of allopurinol is not mediated through low urate levels. It remains to be seen whether allopurinol has a dose-response relationship with cardiovascular events at higher doses.

## Introduction

Gout is the most prevalent inflammatory arthritis, affecting up to 4% of the population in developed countries [1]. Gout attacks can be effectively prevented by use of urate lowering therapy (ULT) [2], such as allopurinol. The aim of ULT is to prevent future attacks of gouty arthritis primarily by decreasing plasma urate level to below 0.36 mmol/l (6.0 mg/dl), which is below the precipitation point of urate at physiological conditions [3].

Allopurinol is the most widely used ULT. In addition to its urate lowering properties, allopurinol has been shown to reduce blood pressure among patients with hypertension [4], increase work capacity in chronic stable angina patients [5], diminish vascular oxidative stress and endothelial dysfunction [6,7]. In addition, allopurinol 300 mg daily or higher has recently been associated with improved cardiovascular outcome [8].

However, the above-mentioned effects on hypertension and work capacity has only been shown with very high doses (> 400 mg/day and > 600 mg/day respectively) [4,5].

The treat-to-target approach (urate level below 0.36 mmol/l) with ULT is developed for gout related symptoms, but whether this approach is also sufficient to achieve a reduction in cardiovascular risk remains to be established. We sought to investigate the effect of achieving target plasma urate with allopurinol on cardiovascular outcomes in a case-control study nested within long-term users of allopurinol.

## Methods

This case-control study nested among long-term users of allopurinol was carried out in Funen County, Denmark, using health care registries furnished by the Danish health authorities. Cases were defined by an Antiplatelet Trialist’s collaboration (APTC) cardiovascular endpoint and controls were without the APTC endpoint. We ascertained whether individuals had achieved target urate level and analyzed the association between in-target urate level and risk of the APTC endpoint.

### Data sources

We linked data from the following four health care registries:

1. The laboratory database of Odense University Hospital is a clinical laboratorial system, which contains information on all blood samples analyzed at every hospital laboratories in the Funen County since November 1992. The coverage includes primary and secondary health care providers for both in- and outpatients. All urate measurements measured at inhabitants in the Funen County during the study period were covered.
2. The Odense University Pharmaco-epidemiological Database (OPED) is a prescription database holding information on redeemed, reimbursed prescriptions for the citizens of Funen County since 1990 [9]. Data included are a full account of the dispensed product and the date of dispensing. The product is described in terms of the defined daily dose (DDD) and the anatomical-therapeutic-chemical (ATC) code [10]. OPED includes a demographic module with information on residency, migration, births and death, which allowed us to account for censoring.
3. The Funen County Patient Administrative System provides hospital discharge diagnosis for the population of Funen County since 1977 for inpatients and since 1989 for outpatients. The diagnoses are encoded according to the International Classification of Diseases 8^th^ revision (ICD8) until January 1994 and ICD10 thereafter.
4. The Danish Register of Causes of Death holds information on all causes of death among Danish citizens, encoded according to the ICD classification system. The National Board of Health established the current register in 1875 [11].

The Record linkage was made possible by a unique personal identifier, assigned to all Danish citizens since 1968 [12]. In Denmark public health authorities provide virtually all health services, which allows true population based epidemiological studies [13].

### Cases and controls

#### Source population

**Fig 1:**
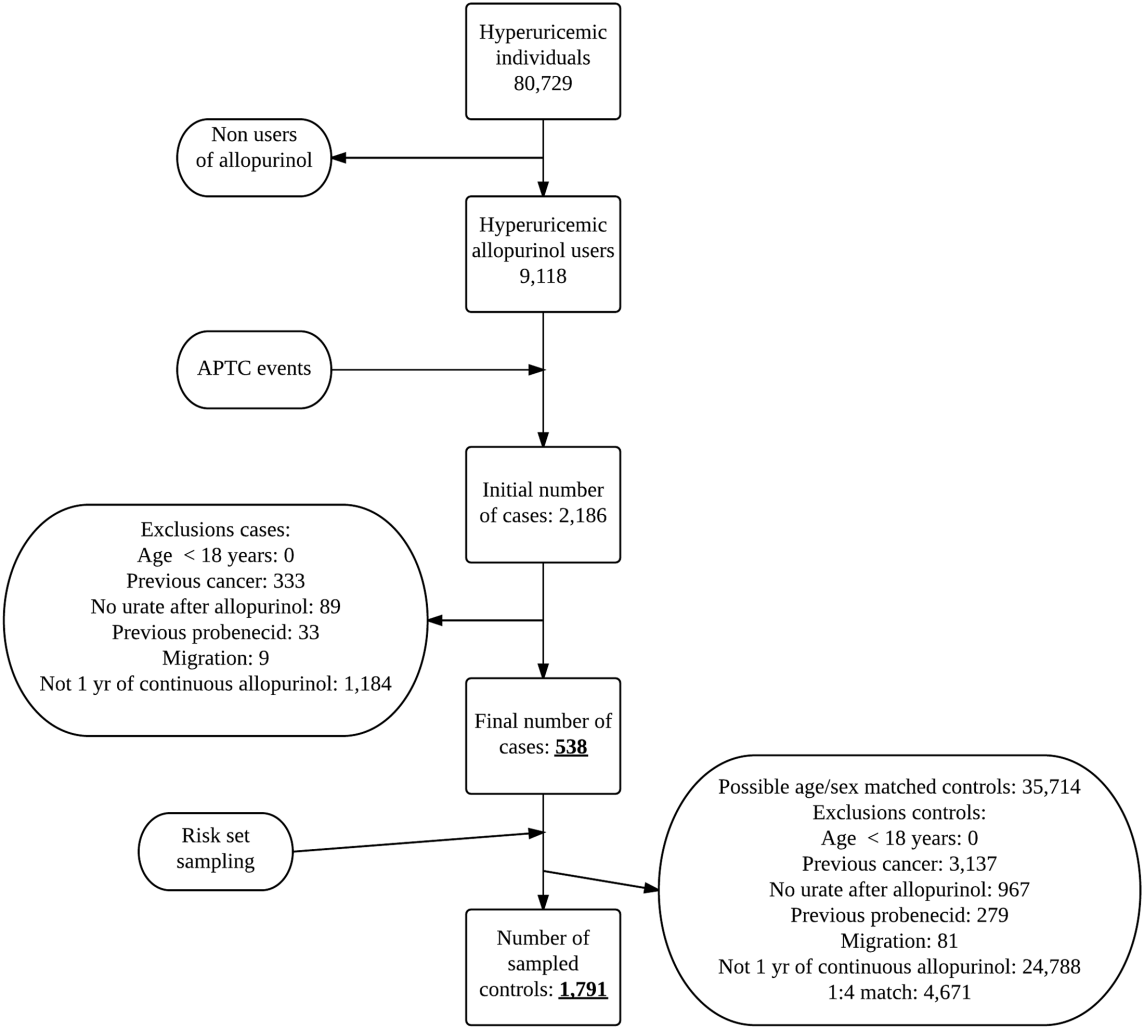
Study flow diagram.

The population used in this setting was derived from the total population from Funen, Denmark (≈ 485,000), during the period of December 1992 through December 2010. Among these we identified first-ever users of allopurinol (ATC M04AA01) with an elevated (≥ 0.36 mmol/l) urate measurement within 2 years prior to their first allopurinol prescription (9,118 individuals) (Fig 1). From this basic cohort basic cohort of hyperuricemic allopurinol users both cases and control were selected.

#### Cases

Cases were identified as individuals developing their first APTC event [14] during follow-up, using the day of this event as the index date. In brief, the APTC endpoint is a non-fatal myocardial infarction (MI) (ICD8 410; ICD10 I21-I22), a non-fatal stroke (ICD8 430–434; ICD10 I60-I64), or cardiovascular death (ICD8 390–458; ICD10 I00-I99) including unknown causes of death (ICD8 780–796; ICD10 R96-R99).

In addition, we applied the following inclusion criteria prior to the index date:

1. At least 18 years of age.
2. Living in Funen at the index date and for the past five years (individuals who migrated inside this period were excluded, see Fig 1.).
3. Cancer free for 5 year, defined by the absence of any of the following diagnosis of malignancies (ICD8 140–207; ICD10 C00-C96), not including non-melanoma skin cancer (ICD8 173; ICD10 C44).
4. No treatment with other ULT than allopurinol.
5. Continuous use of allopurinol in the year preceding the index date. Defined as follows; an individual was considered an allopurinol user if she or he had redeemed prescriptions for allopurinol that added together would cover the index date and the entire year preceding the index date. For each prescription, we assigned a period of usage starting on the date of the prescription and lasting a number of days corresponding to the number of tablets, thus assuming an average daily intake of one tablet. In order to allow for non-compliance and irregular prescription renewals we added a grace period (a period of time in which non-redeeming of allopurinol prescriptions was allowed) of 180 days to the usage period assigned to each prescription of allopurinol. The usage clock was reset at each new prescription.

For numbers of cases excluded in each step and final number of cases see Fig 1.

#### Controls

Controls were selected randomly based on their availability in the cohort of hyperuricemic allopurinol users and matched by age and sex using risk set sampling with a maximum case:control ratio of 1:4. The controls were subjected to the same inclusion criteria as the cases (except the APTC endpoint). As we had full coverage of death and migrations out of the county, we could effectively account for all individuals’ presence in the cohort. The controls were assigned an index date identical to the index date of the corresponding case in the risk set.

Controls were not allowed to have had an APTC endpoint before their selection as controls, but might have one later and thus be re-selected as cases. By this approach, the odds ratio is an unbiased estimate of the incidence rate ratio [15].

Not all cases had 4 possible matches, therefore the final case:control ratio differs from 1:4. The final number of controls and number excluded at each step of inclusion are given in Fig 1.

### Urate level

Both cases and controls were classified as either hyperuricemic or treated-to-target. This classification was based on an average of all urate samples from the time of allopurinol prescription until the index date (weighted by the number of days spent at each different plasma urate level). The plasma urate level at any given day was set to the same value as the closest measurement prior to that day. Individuals would be classified as treated-to-target if the weighted urate level prior to index date were below 0.36 mmol/l.

### Analysis

The adjusted odds ratios (ORs) with 95% confidence intervals (CI) were derived using conditional logistic regression with adjustment for potential confounders. Variables known or suspected to influence cardiovascular diseases were included as potential confounders in the regression model. Current use of other drugs than allopurinol was defined by the redeeming of a prescription for this particular drug less than 120 days before the index date. Previous diagnoses were defined by having an in- or outpatient contact coded with the corresponding ICD8 or ICD10 code. See S1 Table for further details on included variables.

For missing continuous covariates we used median value imputation and missing dichotomous variables were set to absent. In subgroup analyses defined by covariates with imputed data, individuals with missing data were excluded.

Baseline characteristics were presented for cases and controls as well as exposed and unexposed controls (as a proxy for the source population) [16].

### Additional analyses and pre-specified sensitivity analyses

We carried out a range of supplementary analyses. First we used different exposure definitions, by using different urate cut-off values to define the treat-to-target group (urate < 0.20 mmol/l; urate < 0.3 mmol/l; urate < 0.36 mmol/l; urate < 0.42 mmol/l; urate < 0.48 mmol/l), and by grouping individuals according to their decrease in achieved weighted plasma urate relative to the baseline value.

Subsequently we conducted a number of subgroup analyses. We restricted the analysis by strata of age, sex and renal function (defined as chronic kidney disease stages by the National kidney foundation [17]). We also restricted the analysis by only considering individuals with and without diabetes, hypertension and previous APTC events, in order to estimate the effect in groups with different risk of the outcome. Further we analyzed subgroups defined by cumulative doses of allopurinol; (<200 g, 200–400 g, > 400 g) and as average daily intake (< 150 mg/d, 150–250 mg/d and > 250 mg/d).

One critical issue in this study is the definition of when an individual is treated-to-target. First, we performed an analysis were the plasma urate level at any given day was set to the mean value as the closest measurement prior to and after that day, rather than the first value alone. Second, we only included urate measurement from the last 1 and 3 years before the index date to calculate the weighted urate levels. Third, we only considered the latest urate measurement. Fourth, we omitted urate measurements inside 3 month of the index date; this analysis was done to check for any biased results caused by urate levels measured in conjunction with an admission for a case event. Lastly, we changed the grace period for allopurinol prescriptions to 0, 1, 3 and 12 months.

Post hoc we conducted an analysis applying all covariates one by one and in groups (i.e. all comorbidities, all laboratory values or all prescriptions), because the estimates changed markedly after adjustment.

### Ethics

The project was approved by the Danish Data Protection Agency. Registry based studies do not require ethical approval in Denmark, nor do it require personal written consent [18]. All individuals were anonymized prior to retrieval of data and all analyses were performed at anonymized data.

## Results

We identified 538 APTC cases and 1,791 controls with at least one year of continuous allopurinol use. Altogether 1,638 (70.3%) were men and the median age was 78 (IQR; 70–84) years. The median urate levels decreased markedly after allopurinol treatment start among both cases and controls—on average a 27.4% decrease was observed. Other characteristics of the APTC cases and their controls along with exposed and unexposed controls are presented in Table 1.

**Table 1:** Characteristics of APTC cases and their corresponding controls, and for unexposed and exposed controls (proxy for distribution in the source population).

	Cases (n = 538)	Controls (n = 1,791)	Unexposed Controls (n = 1,063)	Exposed Controls (n = 728)
Men	365 (67.8%)	1,273 (71.1%)	815 (76.7%)	458 (62.9%)
Women	173 (32.2%)	518 (28.9%)	248 (23.3%)	270 (37.1%)
Age, median (IQR)	79 (71–86)	77 (70–84)	77 (70–83)	78 (71–85)
History of:				
Ischemic heart disease	284 (52.8%)	589 (32.9%)	390 (36.7%)	199 (27.3%)
Heart failure	241 (44.8%)	393 (21.9%)	271 (25.5%)	122 (16.8%)
Atrial fibrillation	176 (32.7%)	363 (20.3%)	264 (24.8%)	99 (13.6%)
Stroke	101 (18.8%)	249 (13.9%)	158 (14.9%)	91 (12.5%)
Diabetes mellitus	128 (23.8%)	274 (15.3%)	176 (16.6%)	98 (13.5%)
Hypertension	221 (41.1%)	532 (29.7%)	339 (31.9%)	193 (26.5%)
COPD	92 (17.1%)	180 (10.1%)	121 (11.4%)	59 (8.1%)
Charlson comorbidity index:				
0	70 (13.0%)	706 (39.4%)	392 (36.9%)	314 (43.1%)
1	140 (26.0%)	440 (24.6%)	248 (23.3%)	192 (26.4%)
2	105 (19.5%)	266 (14.9%)	166 (15.6%)	100 (13.7%)
≥3	223 (41.4%)	379 (21.2%)	257 (24.2%)	122 (16.8%)
Current drug use (baseline):				
Diabetes-drugs (ever use)	87 (16.2%)	259 (14.5%)	168 (15.8%)	91 (12.5%)
Vitamin K antagonists	71 (13.2%)	206 (11.5%)	166 (15.6%)	40 (5.5%)
ADP-receptor inhibitors	9 (1.7%)	20 (1.1%)	14 (1.3%)	6 (0.8%)
Low-dose ASA	177 (32.9%)	457 (25.5%)	269 (25.3%)	188 (25.8%)
Dipyridamole	37 (6.9%)	86 (4.8%)	45 (4.2%)	41 (5.6%)
Heart glycosides	155 (28.8%)	288 (16.1%)	203 (19.1%)	85 (11.7%)
Nitrate vasodilators	128 (23.8%)	207 (11.6%)	112 (10.5%)	95 (13.0%)
Thiazide diuretics	80 (14.9%)	223 (12.5%)	142 (13.4%)	81 (11.1%)
Loop diuretics	330 (61.3%)	748 (41.8%)	463 (43.6%)	285 (39.1%)
Aldosterone antagonists	99 (18.4%)	172 (9.6%)	124 (11.7%)	48 (6.6%)
Betablockers	159 (29.6%)	506 (28.3%)	332 (31.2%)	174 (23.9%)
Calcium antagonists	128 (23.8%)	426 (23.8%)	243 (22.9%)	183 (25.1%)
RAAS blockers	248 (46.1%)	746 (41.7%)	454 (42.7%)	292 (40.1%)
Statins	114 (21.2%)	451 (25.2%)	292 (27.5%)	159 (21.8%)
COPD related drug use	76 (14.1%)	229 (12.8%)	148 (13.9%)	81 (11.1%)
Systemic corticosteroids	70 (13.0%)	116 (6.5%)	71 (6.7%)	45 (6.2%)
NSAIDs	111 (20.6%)	387 (21.6%)	228 (21.4%)	159 (21.8%)
Blood measurements (baseline):				
Urate level prior to allopurinol, median (IQR)	0.55 (0.49–0.64)	0.52 (0.46–0.60)	0.54 (0.49–0.62)	0.49 (0.43–0.55)
Weighted urate level, median (IQR)	0.39 (0.33–0.47)	0.38 (0.32–0.43)	0.42 (0.39–0.47)	0.31 (0.27–0.34)
Urate < 0.36 mmol/l (treated to target)	197 (36.6%)	728 (40.6%)	0 (0.0%)	728 (100.0%)
eGFR, median (IQR)	43 (31–58)	52 (40–67)	50 (38–65)	55 (43–69)
High HbA1c (> 6.5%)	82 (15.2%)	212 (11.8%)	140 (13.2%)	72 (9.9%)
High cholesterol (> 5 mmol/l)	115 (21.4%)	329 (18.4%)	215 (20.2%)	114 (15.7%)
Proteinuria	40 (7.4%)	61 (3.4%)	49 (4.6%)	12 (1.6%)

IQR = interquartile range; IHD = ischemic heart disease; COPD = chronic obstructive pulmonary disease; DM = diabetes mellitus; ASA = acetylsalicylic acid; ADP = adenosinediphosphat; VKA = vitamin-K-antagonists; RAAS = renin-angiotensin-aldosterone-receptor; NSAID = non-steroidal anti-inflammatory drugs; eGFR = estimated glomerular filtration rate; HbA1c = hemoglobin A1c

### Treat to target approach

Among cases and controls, 197 (36.6%) and 728 (40.6%) were treated-to-target plasma urate level below 0.36 mmol/l respectively. The crude and adjusted odds ratios (OR) of APTC events among individuals treated-to-target urate below 0.36 mmol/l were 0.77 (95% CI 0.62–0.95) and 1.01 (95% CI 0.79–1.28) (Table 2).

**Table 2:** Odds ratios for APTC event of treated-to-target for different subgroups.

Exposure pattern	Cases In target / Not in target	Controls In target / Not in target	Crude OR (95% CI)	Adjusted* OR (95% CI)
All users	197 / 341	728 / 1063	0.77 (0.62–0.95)	1.01 (0.79–1.28)
Sex				
Men	119 / 246	458 / 815	0.81 (0.62–1.04)	1.03 (0.77–1.38)
Women	78 / 95	270 / 248	0.68 (0.47–1.00)	0.97 (0.62–1.53)
Age groups				
Age < 60 years	8 / 32	49 / 99	0.41 (0.16–1.08)	0.45 (0.09–2.36)
Age 60–79 years	86 / 155	356 / 535	0.80 (0.59–1.08)	0.98 (0.69–1.41)
Age ≥ 80 years	103 / 154	323 / 429	0.79 (0.57–1.08)	1.10 (0.76–1.59)
Comorbidities				
Diabetes	44 / 103	141 / 228	0.88 (0.48–1.60)	1.64 (0.47–5.77)
No diabetes	153 / 238	587 / 835	0.88 (0.68–1.14)	1.12 (0.84–1.50)
Hypertension	70 / 151	193 / 339	0.72 (0.45–1.14)	0.99 (0.56–1.72)
No hypertension	127 / 190	535 / 724	0.82 (0.61–1.10)	1.08 (0.75–1.55)
Previous APTC	52 / 106	129 / 232	1.00 (0.53–1.89)	1.11 (0.39–3.15)
No previous APTC	145 / 235	599 / 831	0.75 (0.58–0.96)	0.95 (0.70–1.29)
Renal function				
eGFR > 60 ml / min	48 / 71	290 / 366	0.94 (0.54–1.63)	1.33 (0.60–2.96)
eGFR 30–60 ml / min	107 / 196	369 / 567	0.76 (0.54–1.05)	1.00 (0.68–1.48)
eGFR < 30 ml / min	42 / 74	69 / 130	1.15 (0.47–2.82)	N / A
Cumulated doses of allopurinol (g)				
< 200 g	66 / 195	253 / 540	0.68 (0.44–1.04)	0.97 (0.57–1.65)
200–400 g	72 / 97	238 / 307	0.77 (0.42–1.42)	0.62 (0.16–2.31)
≥ 400 g	59 / 49	237 / 216	0.95 (0.52–1.75)	1.25 (0.23–6.83)
Average mg allopurinol per day (mg)				
< 150 mg	51 / 229	235 / 714	0.63 (0.42–0.95)	0.83 (0.51–1.37)
150–250 mg	62 / 79	201 / 231	0.81 (0.43–1.50)	1.30 (0.36–4.67)
≥ 250 mg	84 / 33	290 / 117	0.84 (0.40–1.79)	4.41 (0.83–23.41)
Duration of allopurinol therapy				
1–2 years	66 / 137	224 / 360	0.73 (0.46–1.18)	0.88 (0.47–1.66)
≥ 3 years	86 / 122	336 / 461	0.88 (0.60–1.29)	1.16 (0.71–1.91)

*) Adjusted by variables presented in S1 Table.

### Allopurinol exposure patterns

We found no association of increasing cumulative amounts of allopurinol with respect to APTC outcomes (Table 2). Similarly, we did not find any association with duration of continuous allopurinol use or with increasing amounts of daily use (Table 2). All tests for trend were statistically non-significant.

### Subgroups

No differences in analysis of different subgroups defined by age, sex or a history of diabetes, hypertension or cardiovascular disease were found (Table 2).

### Urate exposure patterns and cardiovascular events

We found no association between the degree of change in urate level and cardiovascular events. Changing the urate target to different levels showed an increased risk of APTC events for very low levels of urate (< 0.20 mmol/l). Results are presented in Table 3.

**Table 3:** Odds ratios for different exposure definitions of treated-to-target urate levels.

	Cases In target / Not in target	Controls In target / Not in target	Crude OR (95% CI)	Adjusted* OR (95% CI)
Urate threshold for treat-to-target				
< 0.20 mmol/l	16 / 522	22 / 1769	2.17 (1.07–4.43)	2.29 (1.05–4.97)
< 0.30 mmol/l	94 / 444	317 / 1474	0.88 (0.66–1.16)	1.11 (0.81–1.52)
< 0.36 mmol/l	197 / 341	728 / 1063	0.77 (0.62–0.95)	1.01 (0.79–1.28)
< 0.42 mmol/l	326 / 212	1231 / 560	0.63 (0.51–0.78)	0.94 (0.73–1.20)
< 0.48 mmol/l	437 / 101	1559 / 232	0.60 (0.46–0.79)	0.88 (0.65–1.20)
Percent change from baseline urate level				
0–10	508 / 30	1665 / 126	1.27 (0.83–1.94)	1.24 (0.78–1.98)
> 10	467 / 71	1501 / 290	1.18 (0.88–1.58)	1.22 (0.89–1.68)
> 20	387 / 151	1212 / 579	1.15 (0.91–1.44)	1.07 (0.83–1.38)
> 30	268 / 270	794 / 997	1.10 (0.89–1.35)	1.07 (0.85–1.36)
> 40	160 / 378	398 / 1393	1.32 (1.05–1.66)	1.15 (0.88–1.50)
> 50	71 / 467	153 / 1638	1.57 (1.15–2.16)	1.43 (1.00–2.04)
> 60	30 / 508	47 / 1744	2.17 (1.31–3.61)	1.97 (1.13–3.46)

*) Adjusted by variables presented in S1 Table

### Sensitivity analyses

Changing the definition of treated-to-target to include only the last urate measurement or the definition of weighted urate to only include the last 1 and 3 years of urate measurements did not change the overall results (data not shown). Neither did using the alternative approach to calculate the weighted urate levels. Changing the grace periods did not alter the main results, even though there was a non-statistically significant association with lower ORs for the primary endpoint as the grace periods decreased (S2 Table).

The analyses including potential confounders one at a time showed that no single dominating covariate or group of covariates was responsible for the heavy adjustment (S3 Table).

## Discussion

We found no association between treated-to-target urate level and cardiovascular events among long-term (> one year) allopurinol treated individuals. Extensive supplementary analyses failed to identify subgroups or exposure patterns that were plausibly related to a reduced risk of the APTC outcome.

Given the well-established link between hyperuricemia and adverse cardiovascular outcomes, and the extensive literature on beneficial effect of allopurinol on proxies for cardiovascular risk [4,5,7,19], the result may seem surprising. Several counter-hypotheses need to be considered.

One hypothesis is that the effect on cardiovascular risk is mediated through urate lowering but this association takes place at higher allopurinol doses than used for gout treatment. This is supported by evidence suggesting that high doses of allopurinol will result in non competitive inhibition of xanthine oxidase (the rate-limiting enzyme in purine catabolism) [20], and the fact that many gout patients are treated with too low doses of allopurinol [21], which also seems to be the case in this study population with an average allopurinol intake below 140 mg daily.

Another hypothesis would be that the cardiovascular effect of allopurinol is mediated through inhibition of xanthine oxidase rather than the lowering of plasma urate. Xanthine oxidase holds important oxidative and antioxidant properties [22]. In pathological conditions, such as high levels of xanthine, this enzyme produces reactive oxygen species that induces inflammation and cardiovascular diseases [23]. A very steep dose-response relationship between allopurinol and cardiovascular diseases without any direct relation to urate level has been suggested in a study comparing different doses of allopurinol (300 vs. 600 mg / day) showing an improvement on endothelial function with high doses of allopurinol but not low doses, placebo or probenecid [7]. The effect of another xanthine oxidase inhibitor (febuxostat) has in a small study been shown to reduce the arterial stiffness [24]. Unfortunately, other studies have indicated an increased cardiovascular risk among febuxostat treated compared to allopurinol although not statistically significant [25,26].

Finally, our results could be confounded. The use of health care registries means, that we do not have access to information on some important covariates e.g. smoking. Heavy smoking has been associated with increased activity of xanthine oxidase [27], but is in part controlled for, by including surrogate markers of smoking in the regression model (e.g. COPD diagnoses and COPD related prescriptions) and to our knowledge the plasma urate level is not affected by smoking. Information on dietary factors [28] and alcohol consumptions [29], both known to alter urate levels were not available either. Some of these factors are also associated with the metabolic syndrome and increased cholesterol, and might have confounded the results. However, the effect of such confounding is likely limited, as it is widely appreciated that the diet alone will not change urate levels sufficiently in gout patients [30]. Another limitation is the missing information of some intermediate endpoints (e.g. arterial stiffness or coronary calcifications) along the path to develop an APTC outcome. We had prescription data dating back to 1990. Some of our patients may have used allopurinol before then, and their pre-treatment duration of hyperuricemia could therefore not be estimated precisely. As it potentially could affect their pre-treatment cardiovascular risk, this entails a risk of residual confounding. However, we do not have evidence to support that the duration of hyperuricemia affects the ability of allopurinol to reach target urate.

These limitations aside, our study has several strengths. It is based on the entire population in Funen County, with full access to prescription records, admissions, blood samples and migrations. Virtually all health care related contacts in Denmark is provided by the national public health authorities and hence included in this study. Thereby, there is little risk of selection bias in our material. We expect almost complete capture of outcome events. As it is standard practice to admit all individuals with chest pain or dyspnea along with individuals with impaired neurology or symptoms of transitory ischemic attacks, it is highly unlikely that we have missed a substantial number of cases. As for patients who died immediately from the MI or stroke, the causes of death were ascertained from the Danish register of causes of death.

Given the very high validity of cardiovascular discharge diagnoses in Danish registries [31,32] there is little risk of misclassification of outcomes. We based our exposure measure on allopurinol prescriptions and urate measurements and we required the individuals to have been treated for at least a year. We thus find it unlikely that the patient would redeem multiple allopurinol prescriptions with patient co-payment without taking the drug. The urate levels were measured in an accredited laboratory. In all, there is little risk of substantial misclassification of exposure.

We have shown that the extensive adjustment is caused by a”little strokes fell great oak” phenomenon and not by one single covariate, which could have biased the results.

A treat-to-target approach is the mainstay in gout treatment [2,30], but this approach does not seem to confer a better cardiovascular prognosis than an allopurinol treatment failing to reach target. We propose that the cardiovascular effect might not be mediated through urate lowering. At first glance, it may seem as an awkward suggestion, since it is well established that hyperuricemia is associated with high cardiovascular risk, that allopurinol lowers urate levels and that allopurinol improves the cardiovascular risk. However, there are equivalent examples from other parts of the pharmacotherapeutic literature. E.g., the effect of statins on cardiovascular risk is not entirely mediated by cholesterol lowering [33–35], and the effect of ACE-inhibitors on stroke risk is not entirely explained by lowering of blood pressure [36].

It is important to emphasize that our results do not contradict that allopurinol is effective in preventing cardiovascular event. Our result is fully compatible with a dose dependent effect of allopurinol, but for a given dose of allopurinol the effect on urate level is not important for achieving the cardiovascular effect. Future studies will establish the role of allopurinol in cardiovascular prophylaxis and in particular, the nature of its dose-response relationship.

## Supporting Information

S1 Datafile This supporting information file includes the data used for the present study. (XLS)

S1 Table Covariates included in the conditional logistic regression analysis as potential confounders. (DOCX)

S2 Table Main analyses with different grace periods assigned to each allopurinol prescription. (DOCX)

S3 Table Confounder control, main analysis. (DOCX)
