# Supplementary material for: Impact of Urate Level on Cardiovascular Risk in Allopurinol Treated Patients. A Nested Case-Control Study
Source: PLoS One. 2016 Jan 11;11(1):e0146172. doi: 10.1371/journal.pone.0146172 (PMC4709004; doi:10.1371/journal.pone.0146172)
Supplement: S1 Table — (DOCX) [file pone.0146172.s002.docx]

| **Supplementary Table 1. Covariates included in the conditional logistic regression analysis as potential confounders.** | | | |
| --- | --- | --- | --- |
|  | ICD 8 | ICD 10 | ATC-code |
| **Current use of:** |  |  |  |
| Anti-diabetics |  |  | A10 |
| Statins |  |  | C10AA |
| Heart glycosides |  |  | C01A |
| Low-dose ASA |  |  | B01AC06, B01AC30 |
| Vitamin-K antagonists |  |  | B01AA |
| RAAS blockers |  |  | C09 |
| ß-blockers |  |  | C07 |
| Calcium channel blockers |  |  | C08 |
| Loop diuretics |  |  | C03C |
| Thiazide diuretics |  |  | C03A |
| Spironolactone |  |  | C03DA |
| Systemic corticosteroids |  |  | H02AB |
| Nitrate vasodilators |  |  | C01DA |
| ADP receptor inhibitors |  |  | B01AC04, B01AC22, B01AC24 |
| Dipyridamole |  |  | B01AC07, B01AC30 |
| NSAID |  |  | M01A |
| COPD related medicine |  |  | R03BA, R03AC, R03BB, R03AK |
| **Comorbidity:** |  |  |  |
| Diabetes mellitus | 249-250 | E10-14 |  |
| COPD | 490-492 | J44 |  |
| Ischemic stroke or TIA | 432-435 | I63, I64, G45 |  |
| Hypertension | 40 | I10 |  |
| Atrial fibrillation | 4274 | I48 |  |
| Ischemic heart disease | 410-414 | I20-25 |  |
| Heart failure | 4270-4271 | I110, I130, I132, I50 |  |
| Charlson comorbidity index | As described by Charlson, M.E. et al.[1] | | |
| **Blood measurements:** | Inside 6 month prior to index date | | |
| eGFR | Using the Modification of Diet in Renal Disease Study equation.[2] | | |
| HbA1c > 6.5% |  |  |  |
| Total cholesterol > 5 mmol/l |  |  |  |
| Proteinuria (dichotomous) |  |  |  |

Current drug use was defined as redeeming of a prescription within 90 days of index date, besides inhaled drugs, which was defined as ≥ 2 redeemed prescriptions within 180 days of inclusion. Concurrent diseases were defined by ICD8 or ICD10 coded outpatient visits or admissions before index date.

ICD=International Classification of Diseases; ATC=Anatomical Therapeutic Chemical; ASA=Acetylsalicylic acid; RAAS=Renin-angiotensine-aldosterone system; ADP=Adenosine diphosphate; NSAID=Non-steroidal-anti-inflammatory-drugs; COPD=Chronic Obstructive Pulmonary Disease: TIA=Transitory Ischemic Attack; eGFR=estimated Glomerular Filtration Rate; HbA1c=Hemoglobin A1c level.

1. Charlson ME, Pompei P, Ales KL, MacKenzie CR. A new method of classifying prognostic comorbidity in longitudinal studies: development and validation. J Chronic Dis. 1987;40: 373–83.

2. Levey AS, Bosch JP, Lewis JB, Greene T, Rogers N, Roth D. A more accurate method to estimate glomerular filtration rate from serum creatinine: a new prediction equation. Modification of Diet in Renal Disease Study Group. Ann Intern Med. 1999;130: 461–70.
