# Supplementary material for: Impact of Urate Level on Cardiovascular Risk in Allopurinol Treated Patients. A Nested Case-Control Study
Source: PLoS One. 2016 Jan 11;11(1):e0146172. doi: 10.1371/journal.pone.0146172 (PMC4709004; doi:10.1371/journal.pone.0146172)
Supplement: S2 Table — (DOCX) [file pone.0146172.s003.docx]

| **Supplementary Table 2. Main analyses with different grace periods assigned to each allopurinol prescription** | | | | |
| --- | --- | --- | --- | --- |
|  | **Cases**  **In target / not in target** | **Controls**  **In target / not in target** | **Crude OR**  **(95% CI)** | **Adjusted OR (95% CI)** |
| Allopurinol grace period in days between prescriptions |  |  |  |  |
| 0 days | 28 / 56 | 45 / 71 | 0.60 (0.28-1.32) | 0.49 (0.08-2.86) |
| 30 days | 93 / 187 | 308 / 489 | 0.72 (0.52-1.00) | 0.83 (0.57-1.20) |
| 90 days | 155 / 260 | 548 / 776 | 0.74 (0.58-0.95) | 0.98 (0.75-1.30) |
| 360 days | 229 / 413 | 883 / 1346 | 0.79 (0.65-0.96) | 1.00 (0.81-1.24) |
