# Supplementary material for: Impact of Urate Level on Cardiovascular Risk in Allopurinol Treated Patients. A Nested Case-Control Study
Source: PLoS One. 2016 Jan 11;11(1):e0146172. doi: 10.1371/journal.pone.0146172 (PMC4709004; doi:10.1371/journal.pone.0146172)
Supplement: S3 Table — (DOCX) [file pone.0146172.s004.docx]

| **Supplementary Table 3. Confounder control, main analysis** | | | | |
| --- | --- | --- | --- | --- |
| **Adjustments** | **OR main**  (95% CI) | **Prevalence proportion** controls (%) | **OR Outcome** (95% CI) | **OR Exposure** (95% CI) |
| No adjustment | 0.77 (0.62-0.95) | - | - | - |
| Full adjustment | 1.01 (0.79-1.28) | - | - | - |
| Diseases |  |  |  |  |
| All diseases | 0.95 (0.76-1.20) | - | - | - |
| Prev. stroke | 0.77 (0.62-0.95) | 13.9% | 1.49 (1.14-1.94) | 0.85 (0.65-1.12) |
| Diabetes | 0.78 (0.63-0.97) | 15.3% | 1.95 (1.52-2.50) | 0.75 (0.58-0.98) |
| COPD | 0.78 (0.63-0.97) | 10.1% | 1.79 (1.35-2.37) | 0.70 (0.51-0.95) |
| Hypertension | 0.79 (0.64-0.99) | 29.7% | 1.96 (1.57-2.44) | 0.70 (0.57-0.87) |
| Ischemic heart disease | 0.83 (0.67-1.04) | 32.9% | 2.28 (1.84-2.81) | 0.61 (0.50-0.75) |
| Heart failure | 0.88 (0.71-1.10) | 21.9% | 2.93 (2.36-3.65) | 0.54 (0.43-0.68) |
| Atrial fibrillation | 0.84 (0.68-1.05) | 20.3% | 1.99 (1.58-2.50) | 0.47 (0.37-0.60) |
| Drugs |  |  |  |  |
| All drugs | 0.87 (0.70-1.10) | - | - | - |
| Antidiabetics | 0.77 (0.62-0.96) | 14.5% | 1.27 (0.96-1.68) | 0.69 (0.52-0.92) |
| Anticoagulants | 0.78 (0.63-0.97) | 11.5% | 1.31 (0.97-1.78) | 0.41 (0.30-0.57) |
| NSAID | 0.77 (0.62-0.95) | 21.6% | 0.99 (0.77-1.26) | 0.94 (0.74-1.20) |
| Statins | 0.76 (0.62-0.95) | 25.2% | 0.98 (0.76-1.27) | 0.76 (0.60-0.97) |
| Betablockers | 0.77 (0.62-0.96) | 28.3% | 1.21 (0.97-1.51) | 0.75 (0.60-0.94) |
| Heart glycosides | 0.82 (0.66-1.01) | 16.1% | 1.94 (1.52-2.48) | 0.53 (0.41-0.70) |
| ADP inhibitors | 0.77 (0.62-0.95) | 1.1% | 1.55 (0.66-3.66) | 0.79 (0.33-1.90) |
| COPD drugs | 0.77 (0.62-0.95) | 12.8% | 1.14 (0.85-1.53) | 0.73 (0.54-0.98) |
| ASA | 0.76 (0.61-0.94) | 25.5% | 1.67 (1.33-2.10) | 1.04 (0.84-1.30) |
| Dipyridamole | 0.76 (0.61-0.94) | 4.8% | 1.74 (1.15-2.62) | 1.36 (0.89-2.09) |
| Spironolactone | 0.81 (0.65-1.01) | 9.6% | 2.08 (1.57-2.75) | 0.46 (0.33-0.63) |
| Systemic corticosteroids | 0.77 (0.62-0.96) | 6.5% | 2.13 (1.53-2.97) | 0.83 (0.58-1.19) |
| Nitrates | 0.76 (0.62-0.95) | 11.6% | 2.17 (1.67-2.83) | 1.03 (0.78-1.37) |
| Loop-diuretics | 0.84 (0.68-1.05) | 41.8% | 2.21 (1.77-2.77) | 0.56 (0.45-0.69) |
| Thiazide | 0.77 (0.62-0.95) | 12.5% | 1.31 (0.98-1.75) | 0.85 (0.64-1.13) |
| Calcium antagonists | 0.76 (0.62-0.95) | 23.8% | 1.09 (0.86-1.37) | 1.09 (0.87-1.36) |
| Blood measurements |  |  |  |  |
| All blood samples | 0.88 (0.71-1.10) | - | - | - |
| eGFR | 0.86 (0.69-1.07) | - | 0.98 (0.97-0.98) | 1.02 (1.01-1.02) |
| Hemoglobin A1c | 0.77 (0.63-0.96) | 11.8% | 1.46 (1.10-1.95) | 0.72 (0.53-0.97) |
| Cholesterol | 0.78 (0.63-0.96) | 18.4% | 1.38 (1.08-1.76) | 0.70 (0.55-0.90) |
| Proteinuria | 0.79 (0.64-0.98) | 3.4% | 2.64 (1.75-3.98) | 0.45 (0.28-0.74) |
| Charlson Comorbidity index | 0.89 (0.71-1.12) | - | - | - |

**OR main** is the odds ratios associating treated-to-target with APTC events adjusted for the single potential confounder or group of confounders. **Prevalence proportion controls** describes the prevalence proportion of the potential confounder among the controls (proxy for source population). **OR Outcome** describes the association of the potential confounder with the APTC events. **OR Exposure** describes the association of the potential confounder with the exposure (in-target or not).
